# Glycative Stress and Its Defense Machinery Glyoxalase 1 in Renal Pathogenesis

**DOI:** 10.3390/ijms18010174

**Published:** 2017-01-17

**Authors:** Yosuke Hirakawa, Reiko Inagi

**Affiliations:** 1 Division of Nephrology and Endocrinology, The University of Tokyo Graduate School of Medicine, 7-3-1, Hongo, Bunkyo-ku, Tokyo 113-8655, Japan; yohyrakawa-tky@umin.org; 2 Division of Chronic Kidney Disease (CKD) Pathophysiology, The University of Tokyo Graduate School of Medicine, 7-3-1, Hongo, Bunkyo-ku, Tokyo 113-8655, Japan

**Keywords:** chronic kidney disease, AGEs, GLO-1, hypoxia, ER (Endoplasmic Reticulum) stress

## Abstract

Chronic kidney disease is a major public health problem around the world. Because the kidney plays a role in reducing glycative stress, renal dysfunction results in increased glycative stress. In turn, glycative stress, especially that due to advanced glycated end products (AGEs) and their precursors such as reactive carbonyl compounds, exacerbates chronic kidney disease and is related to premature aging in chronic kidney disease, whether caused by diabetes mellitus or otherwise. Factors which hinder a sufficient reduction in glycative stress include the inhibition of anti-glycation enzymes (e.g., GLO-1), as well as pathogenically activated endoplasmic reticulum (ER) stress and hypoxia in the kidney. Promising strategies aimed at halting the vicious cycle between chronic kidney disease and increases in glycative stress include the suppression of AGE accumulation in the body and the enhancement of GLO-1 to strengthen the host defense machinery against glycative stress.

## 1. Introduction

**Figure 1:**
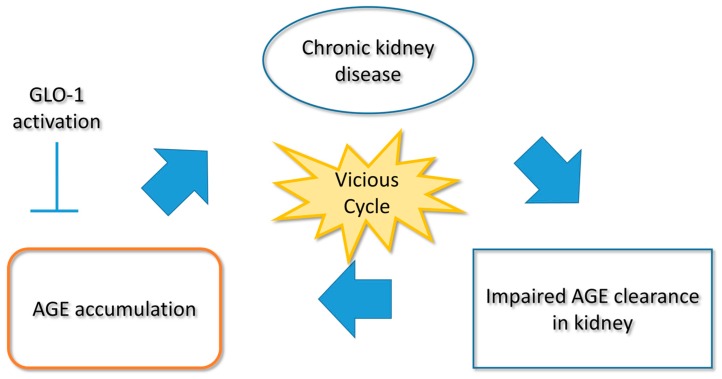
Vicious cycle between CKD and glycative stress. In CKD patients, renal AGE clearance is insufficient because of the low glomerular filtration rate and GLO-1 inhibition. This is followed by AGE accumulation. AGEs themselves have been confirmed to have nephrotoxicity. Thus, CKD and AGE accumulation constitutes a vicious cycle. Abbreviations: AGEs, advanced glycation end products; CKD, chronic kidney disease; GLO-1, glyoxalase 1.

Chronic kidney disease (CKD) is defined as “abnormalities of kidney structure or function, present for over three months, with implications for health”. A diagnosis of CKD is often based on persistent proteinuria and a decreased glomerular filtration rate [1]. The causes and exacerbating factors of CKD are wide-ranging, and include inflammation, hypoxia, endoplasmic reticulum (ER) stress, and glycative stress [2,3,4,5]. With regard to glycative stress, the kidney is the major site of clearance of advanced glycated end products (AGEs), a complex group of compounds formed via glycation of proteins and nucleic acids [6]. However, the kidney is also a target for AGE-mediated organ damage, and any increase in the AGE concentration in the presence of kidney damage thus forms a vicious cycle. This characteristic of AGEs in the context of renal pathogenesis and the progression of CKD has now attracted substantial research attention (Figure 1).

The production of AGEs is thought to be related to senescence. This notion originally arose from research into the effects of glyoxalase 1 (GLO-1) on the lifespan of *C. elegans*. GLO-1 is an enzyme which is part of the glyoxalase system in cytosol, where it prevents the accumulation of AGEs mediated by alpha-oxoaldehydes such as methylglyoxal and glyoxal [7]. GLO-1 over-expression prolongs the lifespan of *C. elegans* by ameliorating mitochondrial protein glycation [8,9]. Following more recent studies, AGEs are now recognized as inducers of premature senescence in both the whole body [10] and the kidney [11].

Another reason for the substantial research interest in AGEs in diabetic kidney disease is that AGEs can be involved in “metabolic memory”, a term used to describe the long-term benefit provided by initial strict glycemic control for a short time. Two clinical trials have shown that initial intensive glucose control is associated with fewer diabetic complications even years after treatment intervention for hyperglycemia ends [12,13]. Since AGE formation is greater in hyperglycemia and AGEs produce adverse long-term results in many organs, AGEs are likely a reason for the occurrence of “metabolic memory” [14]. The existence of “metabolic memory” has changed the clinical management of diabetes mellitus and its complications such as diabetic kidney disease, and the role of AGEs in the pathophysiology of diabetes and diabetic complications has in turn captured worldwide attention.

## 2. Glycative Stress in Pathogenesis of Kidney Disease

### 2.1. Diabetic Kidney Disease

The characteristic increase in glycative stress under high glucose concentrations has made this stress a frequent topic in research into diabetic complications. One convincing proof of the importance of glycative stress in diabetes is that the level of hemoglobin A1c (HbA1c), an Amadori rearrangement compound, is correlated with diabetic complications, and is clinically used as a surrogate marker of an average of blood glucose over approximately one month [15,16,17]. Further evidence is that the level of skin collagen glycation is proven to be correlated to the development of future diabetic complications [18]. Diabetic kidney disease is one of these complications, and its incidence and clinical course are related to the level of HbA1c. Accordingly, the role of AGEs and the glyoxalase system has attracted strong research interest [19].

The enzyme activity of GLO-1 in humans is influenced by at least two polymorphisms in the *GLO-1* gene [20]. The question of whether *GLO-1* polymorphism is related to the onset of diabetes or diabetic complications therefore warrants investigation. An answer might already have been found in the several studies in rats which showed convincing evidence for the close involvement of AGEs and GLO-1 in the pathogenesis of diabetic kidney disease. Initially, Karachalias et al. reported that AGEs accumulate in the glomeruli of streptozotocin-induced diabetic rats [21]. The next question was whether an alteration of GLO-1 activity affects the phenotype of diabetic kidney disease. An answer came with the finding that systemic GLO-1 over-expression decreases tissue AGEs, reduces urinary protein, and ameliorates mesangial expansion in streptozotocin-induced diabetic mice without changing the fasting plasma glucose [22,23,24]. Interestingly, GLO-1 knockdown rats have a similar phenotype to diabetic kidney disease, even without streptozotocin injection [23].

A second approach to evaluating the possible impact of *GLO-1* polymorphism on the onset of diabetes or diabetic complications is via the blocking of AGE accumulation on target organs. Kaida et al. reported that the administration of high-affinity DNA-aptamer raised against AGEs in type 2 diabetic model mice (KKAy/Ta mice) resulted in a reduction in urinary protein, mesangial expansion, and other phenotypes of diabetic kidney disease [25]. These results indicate that AGEs are not only causative agents of diabetic kidney disease but also aggravating factors, at least in mice. Another study emphasized the role of receptor for AGEs (RAGE) in diabetic kidney disease: Reiniger et al. reported that homozygous RAGE knockout ameliorates albuminuria, the reduction in the glomerular filtration rate and other diabetic kidney disease–related renal changes [26].

Several effects of AGEs are known to influence cultured cells originating from nephrons in diabetic models, including podocytes, mesangial cells, and tubular cells. In podocytes and mesangial cells, some histone-modifying proteins are thought to be related to AGE-induced damage. With regard to podocytes, Wang et al. reported that AGEs increase the protein expression of histone deacetylase (HDAC) 4, similarly to high-glucose incubation or the addition of transforming growth factor β (TGF-β) [27]. Wang’s group also showed that in vivo silencing of HDAC4 in the whole kidney attenuated several diabetic changes, including albuminuria, mesangial expansion, an increase in TGF-β, and a reduced nephrin concentration, and that AGEs increase HDAC5 expression in mesangial cells, although the significance of this latter finding is not clear. Another report showed that AGEs decrease the silent information regulator 2-related protein 1 (Sirt1) protein, a histone deacetylase, in mesangial cells, resulting in the promotion of fibronectin 1 and TGF-β1 expression [28]. Taken together, AGE-induced podocytopathy is likely to be related to epigenetics.

With regard to tubular cells, Liu et al. proposed a novel role of AGEs in diabetic kidney disease following their cultured cell experiments. Exposure to AGE-BSA resulted in an increase in autophagosomes, a decrease in autolysosomes, defect in the acidification function in lysosomes, and suppression of the lysosomal degradation function [29]. Given that renal tubular lysosomal dysfunction is detected in type 1 diabetes in both animals and humans [30,31], and that lipid accumulation is seen in diabetic kidney disease [32], this glycative stress–related lysosomal dysfunction appears to be a pathological mechanism in diabetic kidney disease.

### 2.2. Other Non-Diabetic CKD (Chronic Kidney Disease)

A clinical study reported that RAGE polymorphism is related to non-diabetic CKD progression [33]. This finding implicates RAGE pathophysiologically in general CKD as well as in diabetic kidney disease [34]. The binding of AGEs with RAGE is one of the major pathogenic roles of AGEs [35,36], and this finding is thus sufficiently convincing to show that AGEs are related to CKD progression regardless of the blood glucose level even in humans. Indeed, there is a report showing that AGE accumulation and RAGE upregulation were seen in the peritoneal membrane in uremic patients [37]. In this study, the comorbidity of diabetes mellitus was only seen in approximately 20% of the patients.

As for experimental models, Vlassara et al. reported in 1994 that administration of AGE-albumin at 25 mg/kg/day causes glomerulosclerosis and albuminuria without diabetes [38]. In cultured cell experiments, AGEs have been proven to induce epithelial-myofibroblast transdifferentiation in tubular cells [39], an inflammatory response in mesangial cells [40], and podocyte apoptosis [41]. The AGE concentration is increased in non-diabetic CKD [42], and AGE-induced nephrotoxicity therefore plays an aggravating role even in non-diabetic CKD. In turn, the progression of CKD results in decreased renal function, followed by further AGE accumulation owing to decreased AGE clearance in the kidney. Clinical evidence of the relationship between RAGE polymorphism and CKD progression might be related to this vicious cycle; if the affinity between RAGE and AGEs changes, the effect of AGE accumulation on CKD progression may change.

## 3. GLO-1 System in Renal and Vascular Senescence

Since AGEs are closely related to senescence and GLO-1 is a strong detoxifier of AGEs, activating GLO-1 is a promising strategy to avoid renal senescence. Kumagai et al. reported the impact of renal GLO-1 activity in renal ischemia-reperfusion injury (IRI) in rats [43]. After IRI, renal GLO-1 activity was decreased, whereas GLO-1 expression was not changed at the mRNA or protein levels. This result suggests that the decreased activity of GLO-1 in the damaged kidney is caused by some post-transcriptional modification or the existence of inhibitors. Interestingly, the renal damage induced by IRI is ameliorated by over-expression of the human *GLO-1* gene, and accompanied by lower levels of carboxyethyl lysine (CEL), oxidative stress, and apoptosis. A decrease in GLO-1 activity without any change in the protein levels of GLO-1 was also seen in aged rats compared with young rats [44], and thus this result can be interpreted to indicate the prevention of renal senescence induced by IRI via the over-expression of GLO-1. Ikeda et al. indicated that GLO-1 over-expression attenuates renal senescence in aged rats [44]: while cellular indicators of cell senescence such as senescence-associated β-galactosidase (SA-β-gal), p53, p21, and p16 were increased in the kidneys of aged rats, this age-related damage was ameliorated in *GLO-1* transgenic aged rats. This cell-protective effect against cellular senescence is also seen in Human Renal Proximal Tubule Cells (RPTEC) and etoposide-induced cellular senescence models. These results indicate that reduced GLO-1 activity and subsequent renal senescence occur in CKD, and that the activation of GLO-1 can be an encouraging strategy against CKD.

Given that CKD is a high risk factor for cardiovascular disease [45,46], CKD can be interpreted as a systemic vascular disease. Jo-Watanabe et al. identified the role of GLO-1 in age-related endothelial dysfunction [47]. Age-related aortic glycative stress, the subsequent inhibitory phosphorylation of endothelial NO synthase (eNOS) (Thr495), and endothelial dysfunction were alleviated by GLO-1 over-expression. On this basis, the activation of GLO-1 also appears to be a promising way to reduce vascular senescence, which probably exists in CKD patients.

## 4. Renal Hypoxia as the Final Common Pathway of Chronic Kidney Disease

Renal tubulointerstitial hypoxia occurs in CKD; in turn, tubulointerstitial hypoxia exacerbates CKD [3,48]. Our group recently established a direct oxygen measurement technique in living mice, and proved that the kidney was clearly hypoxic in a CKD model [49]. The response against hypoxia is mainly regulated by hypoxia inducible factor (HIF). HIF is composed of two subunits, HIF-α, an oxygen-sensitive protein, and the HIF-β subunit. Three isoforms of HIF-α have been identified, and all of these subunits are degraded in cytosol if sufficient oxygen molecules are present. Once oxygen tension is lowered, HIF-α is accumulated in the cytosol where it forms a heterodimer with HIF-β, which then induces the activation of many HIF-downstream genes. Activation of HIF is thought to be renoprotective, and thus the regulation of HIF has attracted attention.

Recently, Yamaguchi et al. identified a strong association between inflammation and hypoxia [50]. They found that CCAAT/enhancer-binding protein δ (CEBPD) regulates HIF-1 mRNA expression, and that IL-1β, a well-known inflammatory cytokine, induces CEBPD. These results indicate that inflammation activates the HIF pathway. Both hypoxia and inflammation induce reactive oxygen species (ROS), and subsequent oxidative stress. Methylglyoxal (MG) is an AGE precursor. The production of MG increases in oxidative stress [51], and MG is reported to inhibit the heterodimer formation of HIF-α and HIF-β. The idea that the hypoactivation of HIF in renal ischemia underlies the progression of CKD suggests that glycative stress and CKD progression are linked in a vicious cycle, partly via renal hypoxia. More research is needed to elucidate how renal inflammation stands between these two factors.

## 5. Cross-Talk between Glycative Stress, ER (Endoplasmic Reticulum) Stress and Hypoxia

The endoplasmic reticulum (ER) is an organelle which maintains protein homeostasis via posttranscriptional modification, such as folding and degrading unfavorable proteins [5]. ER dysfunction, specifically ER stress, is an important factor in the pathogenesis or progression of various diseases, including kidney disease. ER stress is closely linked to glycative stress. Liu et al. demonstrated one example, showing that the expressions of RAGE, ER stress marker glucose-regulated protein 78 (GRP78), and p21, a cell arrest marker, are all independently correlated with renal senescence, as estimated by SA-β-gal activity, in diabetic kidney disease patients [11]. They also showed that AGEs and ER stress inducers have similar effects on renal tubular cells and result in premature senescence. The link between glycative stress and ER stress has also been shown in other diseases or other organs [52]. This is probably because, since glycation is a non-enzymatic posttranslational modification of proteins, pathogenic glycated proteins may cause protein malfolding, and subsequent ER stress.

It has been shown that ER stress impairs erythropoietin (EPO) production by derangement of the enhancer activity of the EPO gene [53]. Insufficient EPO production or response to EPO is a major mechanism of the anemia experienced by CKD patients, which in turn causes intracellular hypoxia, as shown by our group [49]. AGEs are also related to erythropoietin resistance in CKD patients as uremic toxins [54]. In turn, erythropoietin has a protective effect against AGEs in several cells [55,56,57]. These findings reveal the presence of complicated cross-talk between glycative stress, ER stress, and hypoxia, and therapeutic approaches to CKD must consider these three angles.

## 6. Intervention in Renal Glycative Stress

### 6.1. Activating GLO-1

Since GLO-1 is a powerful detoxifying enzyme of AGE precursors, such as glyoxal (GO) and MG, activation of GLO-1 is a promising strategy against glycative stress–induced organ dysfunction. One mild intervention to activate GLO-1 is the enforcement of exercise. Exercise training in aged rats has multiple effects on glycation stress, including activating GLO-1, reducing MG and CML (GO-adducts) concentrations, and suppressing RAGE expression in the aorta [58]. Against this, another study in rats indicated that exercise increased the numbers of AGE-positive damaged tubular cells [59]; these authors speculated that an increase in the single nephron glomerular filtration rate might result in higher AGE excretion from the blood to primitive urine, and subsequent AGE reabsorption into tubules. Taken together, these findings may suggest that exercise reduces the systemic amount of AGE in the body, but increases AGE accumulation in tubules. Further research to determine whether exercise indeed protects the kidney from AGE-induced injury is warranted. A pharmacological intervention to activate GLO-1 has attracted major interest, and several drugs with this effect have been reported [60,61,62]. Among them, activating the Nrf2-Keap1-ARE (antioxidant responsible elements) pathway was recently reported to have the potential to activate GLO-1 [62,63]. Nrf2 is a transcriptional factor which is regulated by Keap1, and in the presence of stress, such as oxidative stress, the dissociation of Keap1 from Nrf2 increases Nrf2 nuclear accumulation, resulting in its binding to ARE and the subsequent enhancement of downstream gene expressions. Bardoxolone methyl, a drug which activates the Nrf2-Keap1-ARE pathway [64], has been proven to protect kidney function in diabetic kidney disease [65,66,67]. This drug also triggered cardiovascular side effects such as congestive heart failure, and its phase III trial was stopped; however, its renal protective effect (improvement of the estimated glomerular filtration rate) on diabetic kidney disease is clear, and it is again in a phase II trial in Japan, with particular attention being paid to its adverse effects. Data from interim analysis demonstrated a significant improvement in the glomerular filtration rate, as estimated by insulin clearance, without any safety concerns in the bardoxolone methyl group compared to the placebo group. The mechanism by which this drug improves kidney function is not sufficiently understood, but previous research suggests that it may be due to GLO-1 activation, at least in part [62,63]. Another promising GLO-1 inducer is a binary combination of *trans*-resveratrol and hesperetin (tRES-HESP), which synergistically increase GLO-1 expression [68]. tRES-HESP are proven to increase GLO-1 activity, decrease plasma methylglyoxal and total body methylglyoxal-protein glycation, and finally improve metabolic and vascular health in overweight and obese patients [69].

### 6.2. Suppression of AGE Accumulation and Reduction of AGE Production

The suppression of AGE absorption from the intestine is another strategy. The serum AGE concentration is reported to be related to dietary AGE intake [51]. Although one report insists that the influence of the dietary AGE amount is not sufficient to cause vascular endothelial dysfunction in healthy volunteers [70], this report does not clearly mean that the dietary AGE amount does not matter in diabetic or CKD patients. To elucidate the importance of dietary AGE intake in CKD, a high-quality randomized controlled trial is required [71]. To decrease the AGE content, the cooking method is as important as the food being cooked; methods that use lower temperature and higher moisture (i.e., boiling) are desirable because they reduce AGE formation compared with dry heat cooking methods (i.e., broiling) [72].

AGE absorbents can promote the fecal excretion of AGEs. Sevelamer hydrochloride, a frequently used inorganic phosphate binder in dialysis patients, is reportedly able to reduce the plasma pentosidine level in hemodialytic patients [73]. Similarly, this agent also reduces serum levels of MG and (ε)*N*-carboxymethyl-lysine in non-dialytic diabetic patients [74]. Given also that sevelamer is not absorbed into the blood from the gut and can bind to AGE-BSA, these effects are derived from the absorption and fecal excretion of AGEs by sevelamer. Subsequent research revealed that sevelamer carbonate ameliorates albuminuria and reduces circulating and cellular AGEs in type 2 diabetes patients [75]. These reports indicated that such absorption agents will be beneficial to patients with diabetes, especially those with diabetic kidney disease.

Another method of reducing the plasma and renal AGE levels is suppression of AGE formation in the body. Aminoguanidine is a prototype therapeutic agent which scavenges dicarbonyl glycating agents, such as methylglyoxal and glyoxal [76]. However, its clinical trial against diabetic nephropathy in type 2 diabetes mellitus needs to be terminated according to safety concerns and an apparent lack of efficacy.

Several derivatives of vitamin B_1_ and B_6_ have the potential to inhibit AGE formation; the representative drugs are benfotiamine and pyridoxamine, a form of vitamin B_6_ [77,78]. Benfotiamine activates transketolase and has antioxidative effects in diabetes. This drug is proven to protect tissue from high-glucose–induced cell damage via alleviation of AGE accumulation [79,80,81]. Pyridoxamine inhibits AGE formation in vitro [82], and inhibits albuminuria in streptozotocin-induced diabetic rats [83]. A recent report by Golegaonkar et al. indicated that rifampicin, an old antibiotic, has the potential to suppress age-related glycative stress and elongate the lifespan of *C. elegans* [84].

## 7. Future Directions

As described above, AGEs and the kidney are closely related in that AGEs accumulate with renal dysfunction and have a nephropathic role. Although the mechanism of AGE-induced renal damage is quite complicated, the fact that AGEs damage the kidney has been well proven in animal experiments and cells derived from humans. However, clinical research which proves that AGEs are toxic to the kidney or that anti-AGE treatment is effective against CKD progression is not presently sufficient. It is fortunate that nephrologists can easily obtain urine, which is strongly affected by renal conditions, from CKD patients. Together with recent advances in metabolomics analysis, the role of AGEs in clinical settings must be more thoroughly researched.
